# Supplementary figures and images for: Neoblast-like stem cells of Fasciola hepatica
Source: PLoS Pathog. 2024 May 28;20(5):e1011903. doi: 10.1371/journal.ppat.1011903 (PMC11161113; doi:10.1371/journal.ppat.1011903)

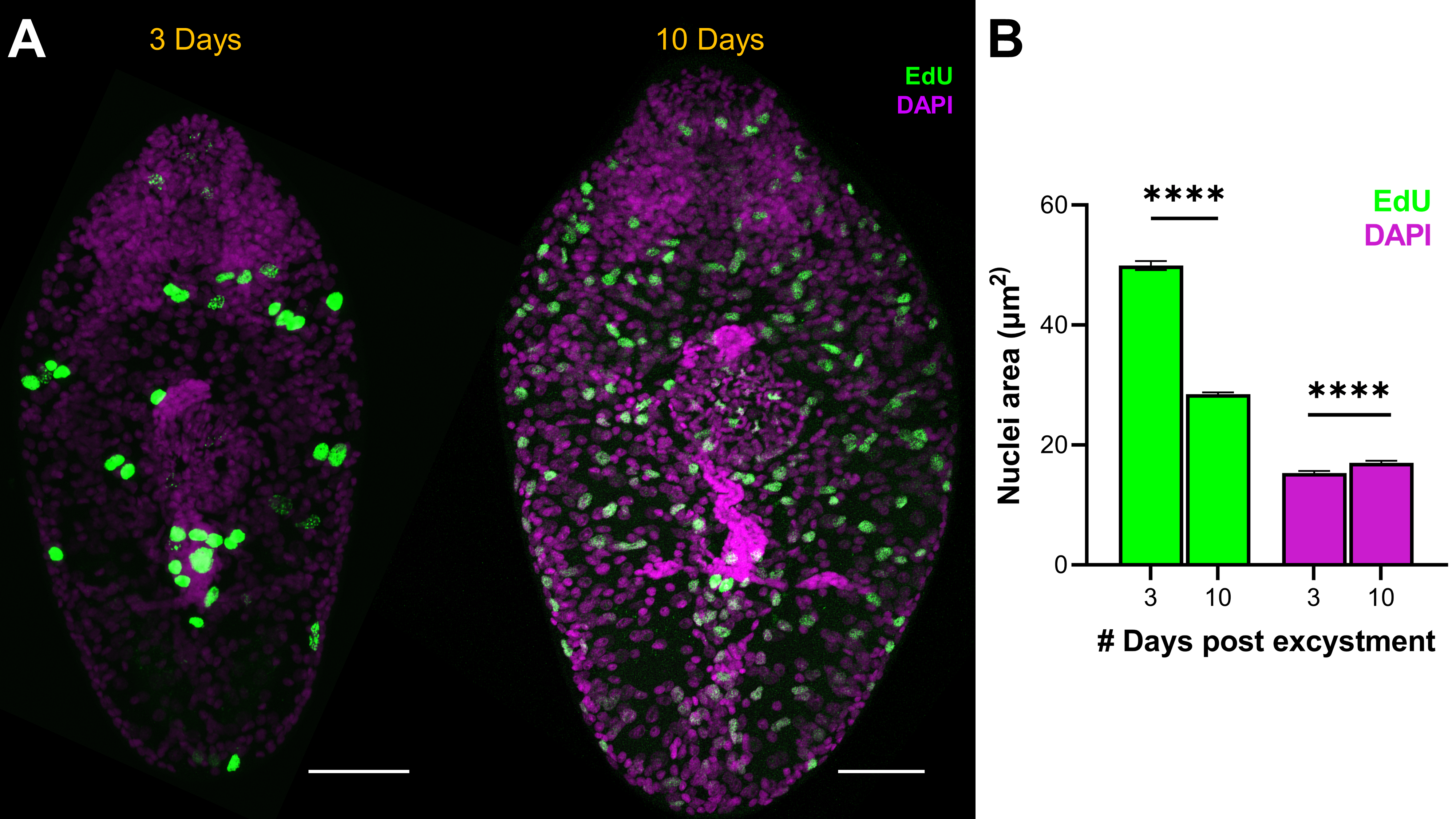

Supplement: S1 Fig — (A) Images of juvenile F. hepatica cultured for three or ten days in CS50 growth inducing media with labelling of proliferative cells (EdU, green) and all nuclei (DAPI, purple); scale bars = 50 μm. (B) Comparison of EdU+ nuclei and EdU- nuclei area in different aged juveniles shows that EdU+ cells are reduced in size by around half as worms age, whereas EdU- cells increase slightly in size (Mann-Whitney U test). ****, p<0.0001. (TIF) [file ppat.1011903.s001.tif]

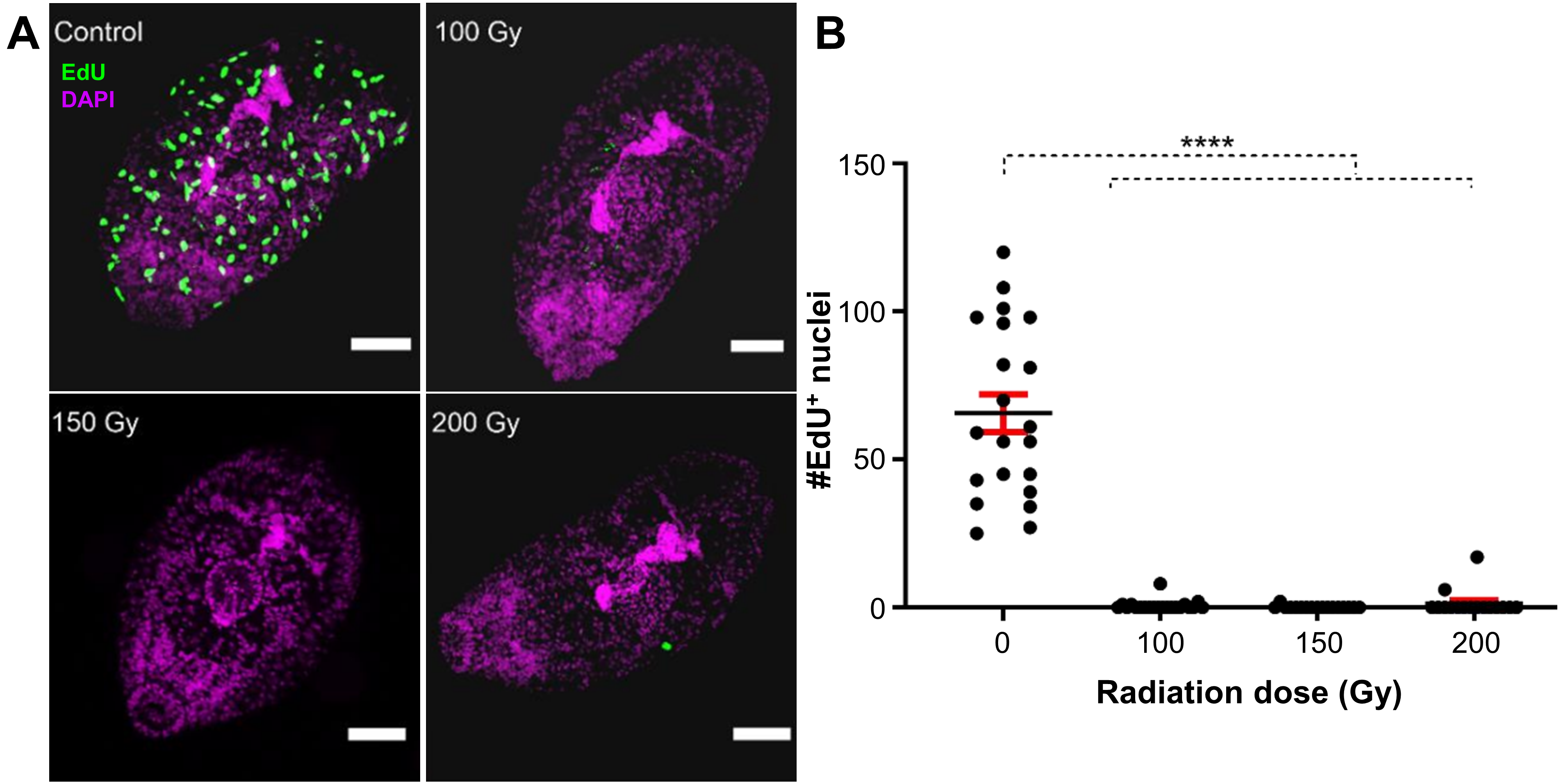

Supplement: S2 Fig — (A) Confocal images of juvenile F. hepatica that were cultured for three days in vitro before being dosed with 0–200 Gy radiation, then cultured for a further 72 h (final 24 h in EdU) and stained for EdU (green) shows that radiation ablates EdU+ nuclei; DAPI (magenta), scale bars = 50 μm. (B) # EdU+ nuclei in juvenile F. hepatica that were cultured for three days in vitro before being dosed with 0–200 Gy radiation shows significant reduction in number of EdU+ nuclei at 100–200 Gy (Mann-Whitney U test). ****, p<0.0001. (TIF) [file ppat.1011903.s002.tif]

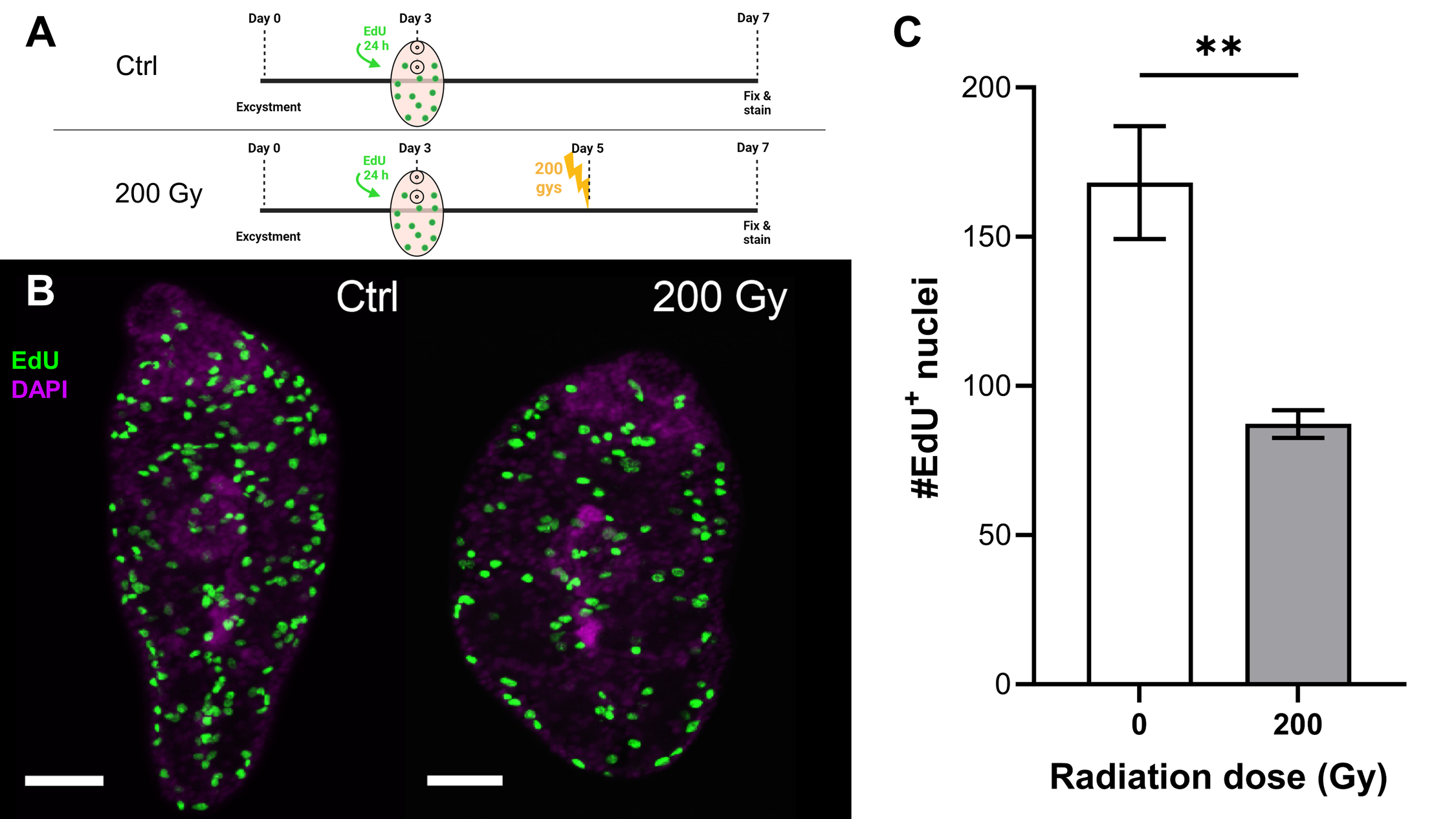

Supplement: S3 Fig — (A) Timeline of experiment showing EdU incubation/labelling prior to irradiation. (B) Confocal images of in vitro juvenile F. hepatica treated according to timeline outlined above shows that cells already labelled with EdU (green) are not completely ablated by 200 Gy of radiation; DAPI counterstain (magenta). (C) # EdU+ nuclei in juvenile F. hepatica treated according to timeline outlined above shows that a significant number of EdU nuclei labelled prior to irradiation are ablated, though not all (Unpaired t test; n = 26). **, p<0.01. Schematic figure created with BioRender.com. (TIF) [file ppat.1011903.s003.tif]

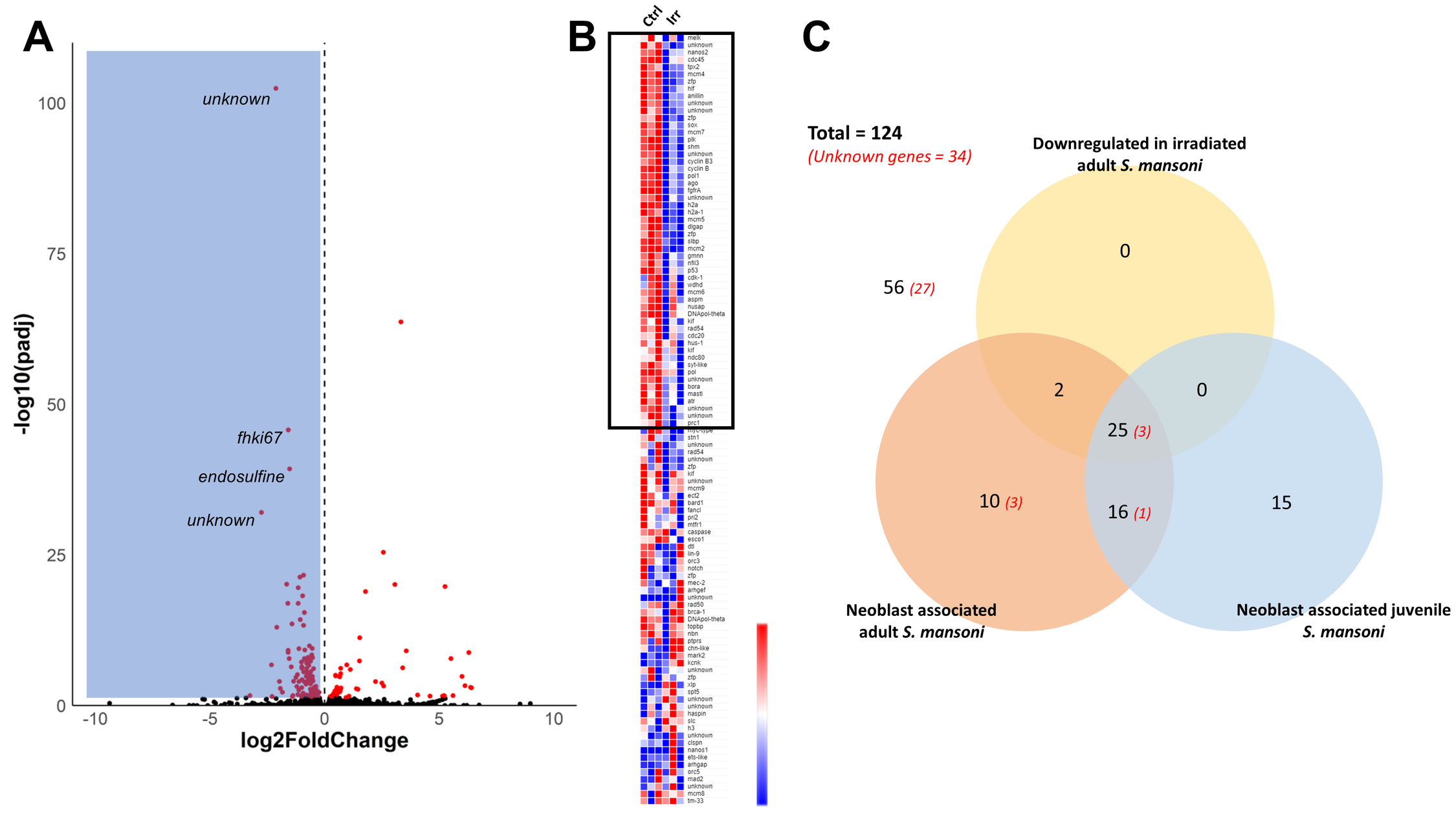

Supplement: S4 Fig — (A)–Volcano plot showing differentially expressed genes (red) following irradiation of juvenile F. hepatica where blue shading indicates downregulated genes likely associated with the ablated neoblast-like genes (greatest DEGs labelled with putative names). (B) Heatmap of expression (max z score, red; min z score, blue) in control and irradiated juvenile F. hepatica for the genes that are homologues of downregulated genes in adult S. mansoni following irradiation. (C) Venn diagram showing occurrence of homologues of F. hepatica downregulated genes following irradiation in various S. mansoni bioinformatic resources: downregulated in irradiated S. mansoni adults; enriched in adult stem cells (single cell); enriched in schistosomule stem cells (single cell). (TIF) [file ppat.1011903.s004.tif]
